# Association between prenatal or postpartum exposure to tobacco smoking and allergic rhinitis in the offspring: An updated meta-analysis of nine cohort studies

**DOI:** 10.18332/tid/146905

**Published:** 2022-04-11

**Authors:** Xinrong Li, Ran Jing, Shenglan Feng, Hui Zhang, Jianfeng Zhang, Jiulin Li, Wencan Cao, Mingjun Jiang, Yang Liu

**Affiliations:** 1 Hospital of Chengdu University of Traditional Chinese Medicine, Chengdu, China

**Keywords:** tobacco, allergic rhinitis, prenatal, postnatal, meta-analysis

## Abstract

**INTRODUCTION:**

Previous studies have suggested an association between tobacco smoke exposure and allergic rhinitis. This study aimed to investigate if prenatal or postpartum smoke exposure will increase the risk of allergic rhinitis in offspring.

**METHODS:**

PubMed, EMBASE, and the Cochrane library were searched from inception to July 2020 for eligible studies investigating the association between smoking exposure and allergic rhinitis. The random-effects model was adopted for the meta-analysis to obtain the summary odds ratio (OR) with a 95% confidence interval (CI). Subgroup analysis based on the age of children was performed. Sensitivity analysis was carried out to check the robustness of the results. Publication bias of included studies was assessed.

**RESULTS:**

This meta-analysis included nine studies, in which six studies suggested that children exposed to prenatal smoking were more likely to develop allergic rhinitis compared with children who were never exposed (OR=1.12; 95% CI: 1.04–1.21). The subgroup analysis divided children those aged <10 years (OR=1.15; 95% CI: 1.06–1.25) and those aged >10 years (OR=0.99; 95% CI: 0.82–1.20). This meta-analysis revealed a positive relationship between postpartum smoke exposure and the development of allergic rhinitis in offspring (OR=1.19; 95% CI: 1.03–1.39) with marked heterogeneity. The subgroup analysis of age in the postnatal group showed similar results in children aged >10 years (OR=1.17; 95% CI: 1.05–1.30) and children aged <10 years (OR=1.21; 95% CI: 0.91–1.60).

**CONCLUSIONS:**

This meta-analysis observed an association between parental smoking exposure and allergic rhinitis in offspring. Our findings indicated that both prenatal and postnatal smoke exposure might be risk factors for allergic rhinitis in the offspring.

## INTRODUCTION

Allergic rhinitis, the most common form of atopic disease, involves 400 million people worldwide and affects people of all ages, peaking in the teenage years, which was especially of high prevalence in industrialized nations^1,2^. The prevalence of allergic rhinitis ranges between 5% and 22%, and there is an increasing trend for the prevalence of allergic rhinitis in China^3,4^. Allergic rhinitis is characterized by pruritus, sneezing, rhinorrhea, and nasal congestion mediated by early-phase and late-phase hypersensitivity responses to indoor and outdoor environmental allergens^5^.

Tobacco smoke exposure especially can induce overexpression of the Toll-like receptor (TLR) and modifies the lipopolysaccharide-mediated responses, the activation of nuclear factor-kB, the release of interleukin-8 by innate lymphoid cell-2 through the production of epithelial cytokines, and the chemotactic activity toward neutrophils^6,7^. Maternal smoking during pregnancy causes epigenetic changes such as overexpression of miRNA-233 and reduces Treg cell numbers in offspring’s cord blood at birth which can result in atopic tendency and succedent risk that persists in early infants^6^. In addition, smoking may facilitate IgE-mediated sensitization and increase allergen-specific IgE in nasal lavage fluid when sensitized allergic patients are exposed to respiratory allergens^8,9^. The IgE antibodies are carried by the blood, and they bind to high-affinity receptors on mast cell membranes. Once re-exposure to allergens happens, IgE on the receptors is cross-linked, and mast cells’ active compounds including cytokines, leukotrienes, prostaglandins, and histamine, could cause an allergic reaction and subsequent risk of rhinitis attack^10^. Passive smoking can exacerbate oxidative stress and arterial dysfunction in children with allergic rhinitis^11^.

Recent cross-sectional studies and meta-analysis suggest an association between parents’ smoking behavior and allergic rhinitis^12,13^. However, the previous meta-analysis failed to assess the effect of prenatal and postpartum smoking on allergic rhinitis in their offspring^13^. Furthermore, most studies included in the meta-analysis were cross-sectional, which do not allow for causal inference and might overestimate relative risks^13^. Therefore, we conducted this meta-analysis to investigate the association between prenatal and postpartum exposure to smoking and allergic rhinitis in offspring and further explore the effects of smoking exposure time and age on allergic rhinitis development.

## METHODS

### Search strategy

The study followed the meta-analysis of observational studies in epidemiology (MOOSE)^14^ and the preferred reporting items for systematic reviews and meta-analyses (PRISMA)^15^. Biomedical databases, including Medline (via PubMed), Embase, and Cochrane library, were searched from inception to July 2020. Both MeSH and free text terms were combined, and the key words included: ‘smoke’, ‘tobacco use’, ‘rhinitis’, ‘allergic’, ‘maternal’, ‘parents’, ‘father’, and ‘child’. The search results were limited to humans, which were adjusted to comply with the relevant rules in each database (Supplementary file Table S1).

### Eligibility criteria

Studies were considered eligible if they: 1) were cohort studies investigated that association between smoking exposure and allergic rhinitis; 2) included endpoints of allergic rhinitis; 3) provided information on any of the prespecified points, including odds ratio (OR) and confidence interval (CI); and 4) were published in the English language. The latest study was included if studies with replicated data or the same population were reported in more than one study. The selection process was divided into three successive stages: title, abstract and full-text selection. Two authors (LL and WC) independently evaluated study titles, abstracts, and full-texts, and disagreements were resolved by discussion or by another investigator (YL).

### Data extraction and quality of evidence

Two authors (RJ and SF) independently extracted data from the eligible studies using a standardized form and were transcribed onto a dedicated database. Any discrepancies were discussed by all authors and resolved by consensus. The data extracted from each report included: 1) study characteristics: authors, year, country, and sample size; 2) exposure parameters: age of children when endpoint assessed, sources of exposure (prenatal or postpartum smoke exposure); and 3) outcome measures: odds ratios of rhinitis from prenatal and postpartum smoking, potential adjusted variables.

The methodological quality of the included cohort studies was quantitatively evaluated according to the Newcastle–Ottawa Scale (NOS) in terms of selection (maximal score of 4), comparability (maximal score of 2), and outcome (maximal score of 3)^16^. Studies scoring >6 were defined as high quality. Disagreements were resolved by discussion.

### Statistical analysis

As a principal summary measure, pooled odds ratios (ORs) and 95% confidence intervals (CIs) were calculated from each study. The crude OR was estimated based on the number of cases in each cohort if it was not reported. Heterogeneity was examined by using Cochran’s Q-statistic. In addition, the I^2^ -test (range: 0–100%) was used to quantify heterogeneity. The random-effects model was used to pool estimates due to a heterogeneous population. Subgroup analysis was conducted to determine the effect of age and adjustment of variables. Sequentially excluding a single cohort study, sensitivity analysis was carried out to check the robustness of the results. Publication bias^17^ was not assessed via Egger’s test due to the limited number of studies included (<10), whereas funnel plots were used to help visualize how the studies were distributed. All analyses were performed using Stata (version 14.0, StataCorp LP, College Station, TX, USA).

## RESULTS

### Study and patient characteristics

**Figure 1:**
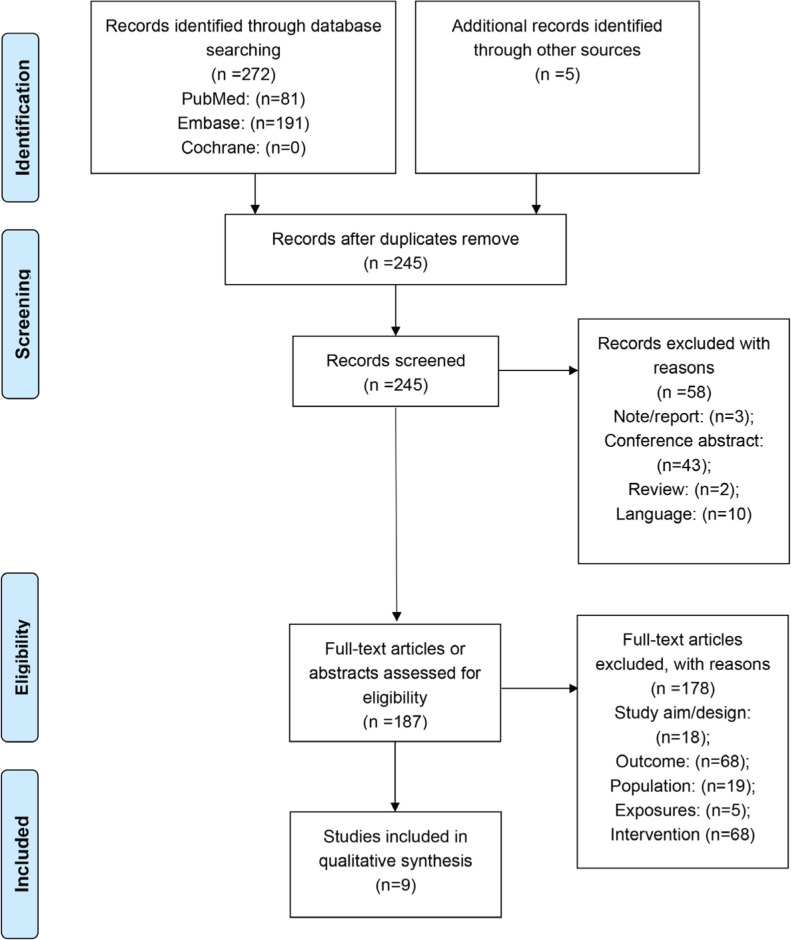
Flowchart of article selection

The flowchart of study selection is shown in Figure 1. The literature search identified 272 publications. In addition, 5 records were identified through manual reference retrieving. After removing duplicates, 245 records were screened according to the inclusion criteria. After initial screening, 58 studies were excluded based on abstract, and 178 studies were excluded after applying the exclusion criteria on the full-text studies. Therefore, 9 studies were finally included in this meta-analysis^12,18-25^, which included 145934 patients. There was one study from USA, Denmark, and India, and two studies from Germany, Sweden, United of Kingdom, respectively. The range of publication year was 2000–2018. Among them, 4 studies investigated children exposed to maternal smoke, 5 studies focused on children exposed to paternal smoke, and 1 study paid attention to parental smoke exposure. The age of children varied from 3 to 18 years. The OR value was reported in 4 studies without adjustment (Supplementary file Table 3). The assessment of the risk of bias is presented in Supplementary file Table 1. The cohort studies included in this meta-analysis were of high quality, all of them scored ≥7 evaluated by NOS criteria (Supplementary file Table 2).

### Effect of prenatal smoke exposure on allergic rhinitis

**Figure 2:**
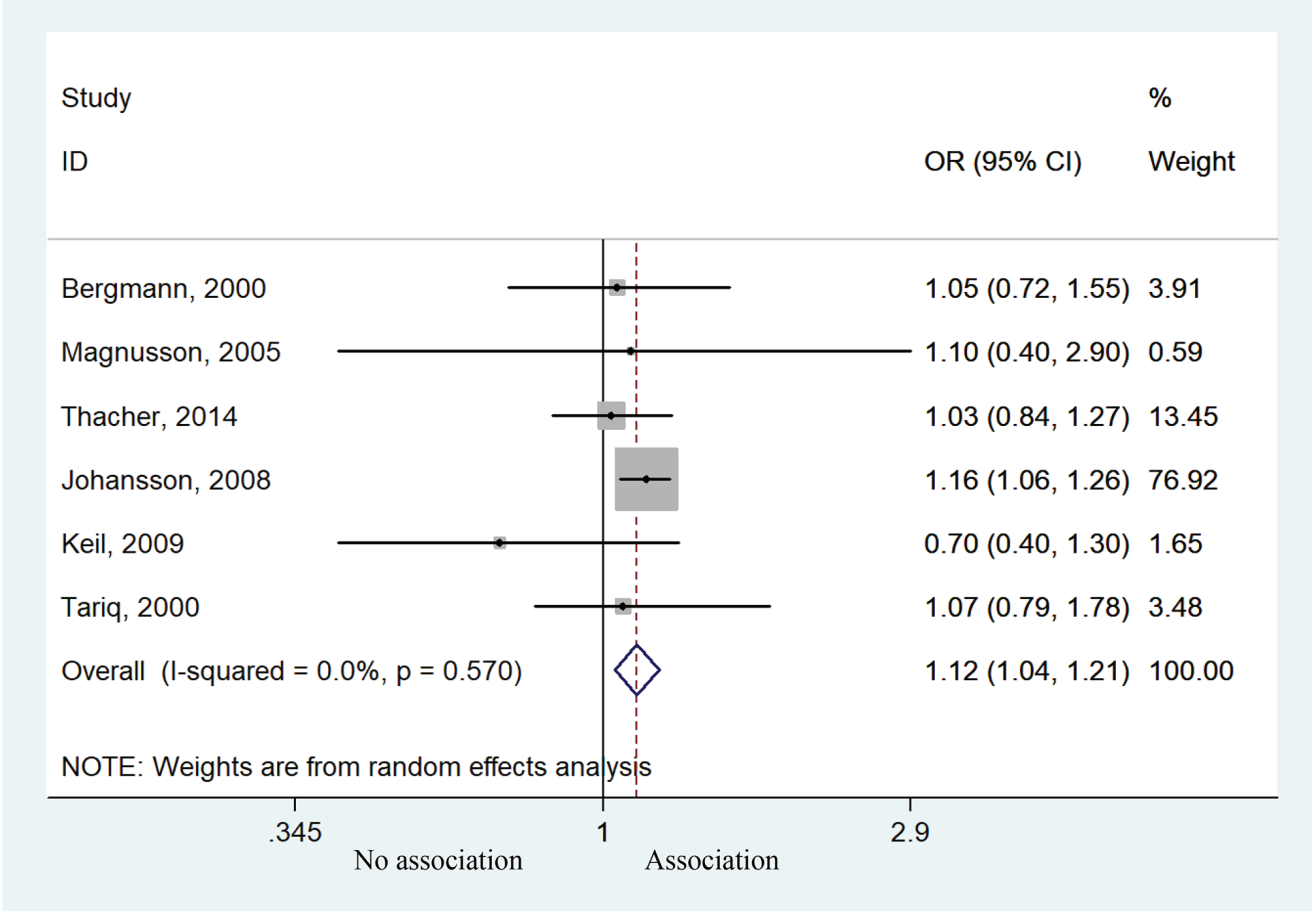
Forest plot of the association between allergic rhinitis and prenatal smoke exposure

**Figure 3:**
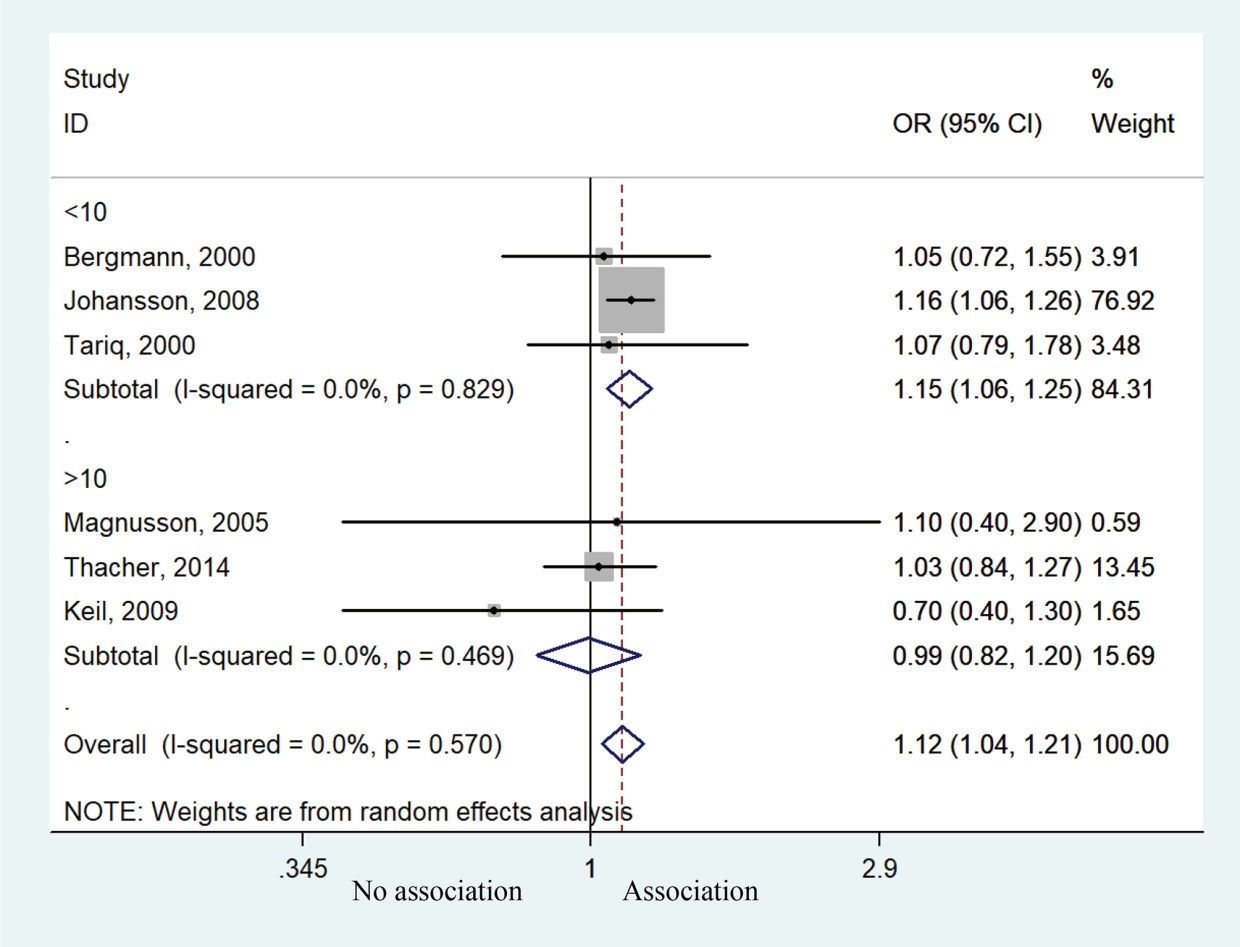
Forest plot of the association between allergic rhinitis and prenatal smoke exposure stratified by age

Six studies contributed to the meta-analysis by analyzing the relationship between prenatal smoke exposure and the risk of allergic rhinitis. Children exposed to prenatal smoking behavior were more likely to develop allergic rhinitis compared with unexposed children (OR=1.12; 95% CI: 1.04–1.21, p=0.002). Heterogeneity was not presented (I^2^=0, p=0.570) (Figure 2). Subgroup analysis of age suggested that children <10 years of age had higher risk of allergic rhinitis (OR=1.15; 95% CI: 1.06–1.25, p=0.001), but those aged >10 years experienced no such effect (OR=0.99; 95% CI: 0.82–1.20, p=0.929) (Figure 3). Sensitivity analysis suggested that the study by Johansson et al.^20^ might affect the association between prenatal smoke exposure and risk of allergic rhinitis (Supplementary file Figure 1).

### Effect of postnatal smoke exposure on allergic rhinitis

**Figure 4:**
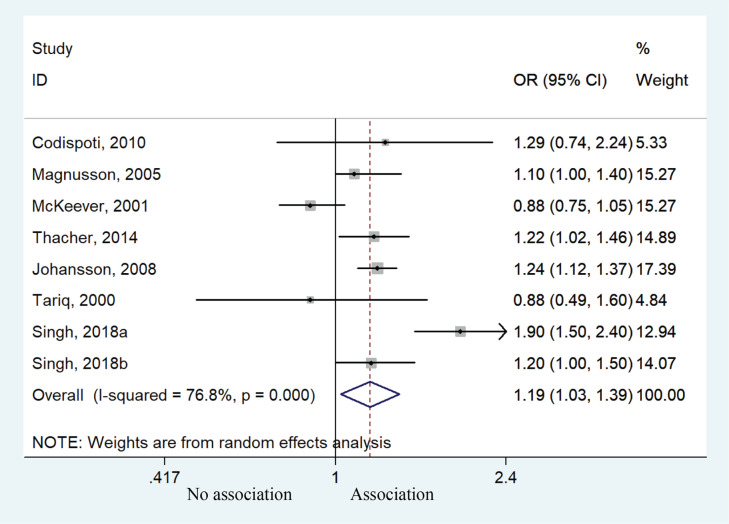
Forest plot of the association between allergic rhinitis and postpartum smoke exposure

**Figure 5:**
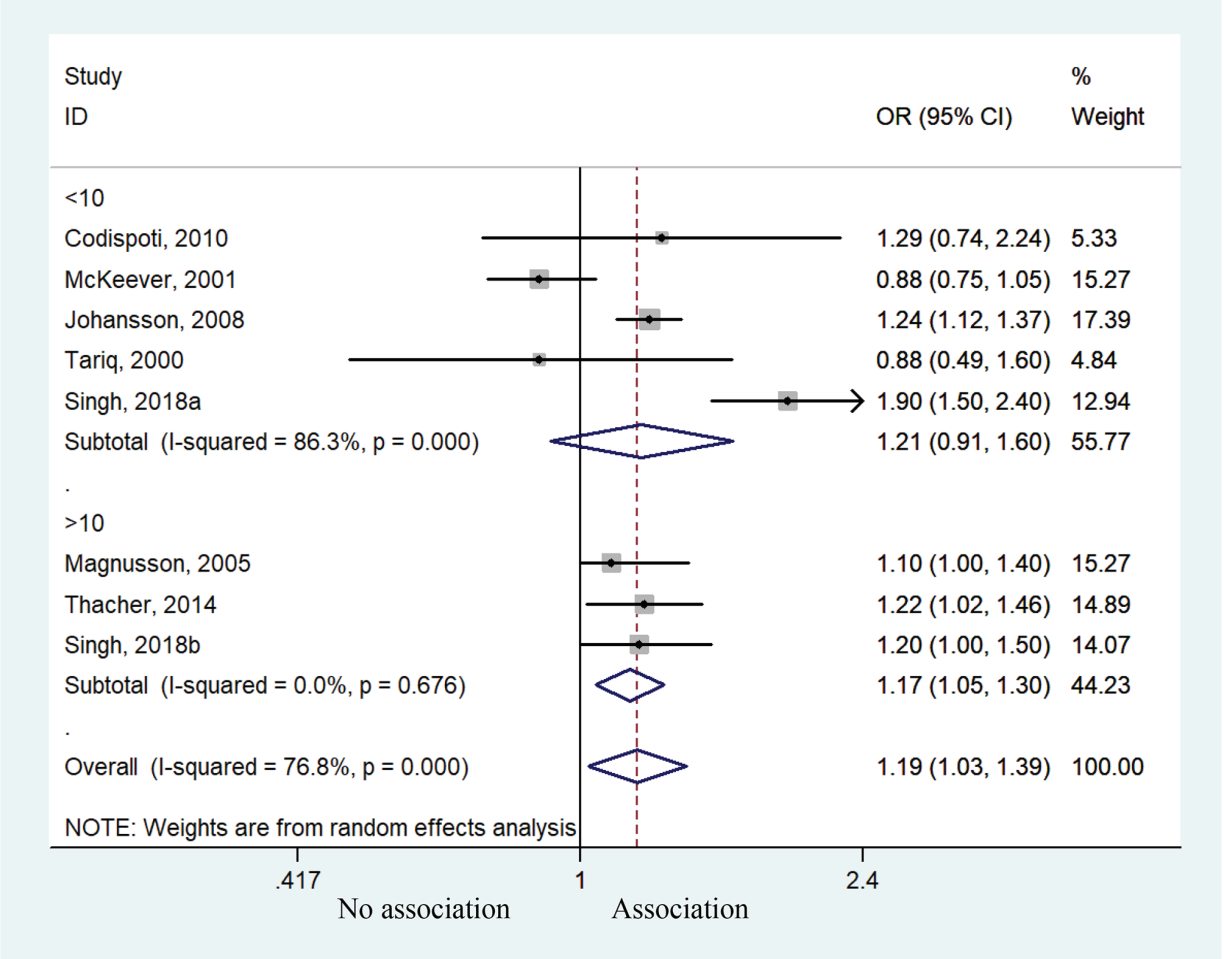
Forest plot of the association between allergic rhinitis and postpartum smoke exposure stratified by age

Eight cohorts studies were available for the meta-analysis, providing an assessment of the association between postpartum smoking behavior and risk of allergic rhinitis. Children exposed to postpartum smoking behavior had a higher risk of developing allergic rhinitis compared to unexposed children (OR=1.19; 95% CI: 1.03–1.39, p=0.021). Heterogeneity was observed (I^2^=76.8%, p<0.001) (Figure 4). Subgroup analysis of age also suggested that children aged >10 years were predisposed to higher risk of allergic rhinitis (OR=1.17; 95% CI: 1.05–1.30, p=0.004). No significant such effect was observed for those aged <10 years (OR=1.21; 95% CI: 0.91–1.60, p=0.186) (Figure 5), but the results might suggest a susceptible tendency for these children to develop allergic rhinitis.

**Figure 6:**
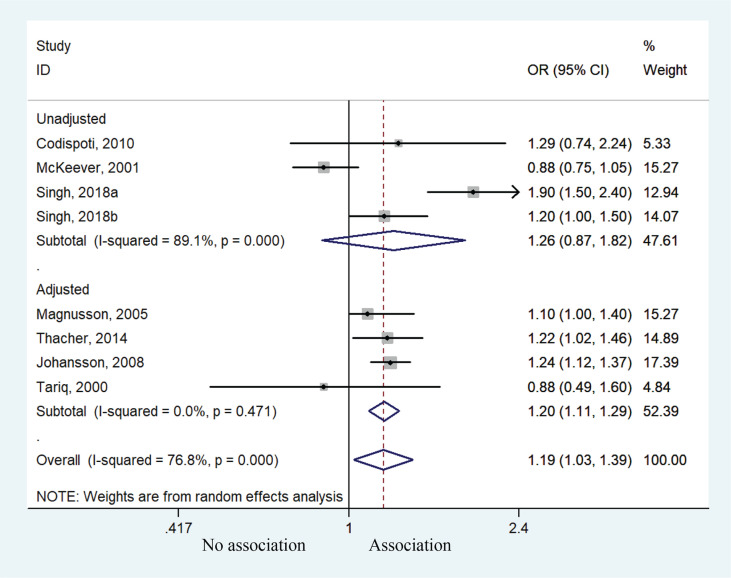
Forest plot of the association between allergic rhinitis and postpartum smoke exposure stratified by covariates adjustments

Subgroup analysis after variable adjustment indicated that children exposed to postpartum smoking behavior had a higher risk of allergic rhinitis with adjustment (OR=1.20; 95% CI: 1.11–1.29, p<0.001), but no significant such effect was found without adjustment of potential confounders even though a similar tendency was noted (OR=1.26; 95% CI: 0.87–1.82, p=0.220) (Figure 6).

Sensitivity analysis confirmed the robustness of the results for the association between postpartum smoking behavior and risk of allergic rhinitis (Supplementary file Figure 2).

### Publication bias

Funnel plots that assess the publication bias in the association between allergic rhinitis and both prenatal and postpartum smoke exposure indicated a relatively balanced distribution (Supplementary file Figures 3 and 4).

## DISCUSSION

This meta-analysis demonstrated that prenatal and postnatal smoke exposure were found to have an effect on the risk of developing allergic rhinitis. Both prenatal smoking of the mother and postnatal smoking of children increased additional threats to the child, with each time frame of exposure adding an additional risk towards the development of allergic rhinitis. Therefore, parents should be educated about the nature of allergic rhinitis, and the increased likelihood of diseases with smoke exposure. Compared with a previous meta-analysis^13^, this study not only observed a significant association between smoking and allergic rhinitis, but also determined the effect of exposure time and age of onset. The attempt to separate children based on prenatal and postpartum exposure and whether they were aged >10 years indicated that exposure in different periods of life might be related to different adverse effects in children with different ages.

The sensitivity analysis of prenatal smoke exposure suggested that the study by Johansson et al.^20^ might affect the association between prenatal smoke exposure and risk of allergic rhinitis. Johansson et al.^20^ reported that children exposed both prenatally and postnatally and those exposed only postnatally had more rhinitis compared with children with non-smoking parents, but they did not show that prenatal tobacco smoke exposure or exposure in early childhood was of significant importance for rhinitis. The reasons accounting for the non-significant results might be due to differences in sample size (n=149 vs n=895) and socioeconomic factors between children with only fetal exposure and those only exposed in early childhood. In addition, the ‘healthy smoker effect’ might also be a contributing factor^26^. Namely, parents of young children who showed these symptoms stopped smoking before the child was 3 years old due to increased knowledge of the impact of environmental tobacco smoke exposure on rhinitis symptoms in the general population. Therefore, it is important to consider the changes in smoke exposure over time.

This meta-analysis comprising only cohort studies was powered to support the causal inference between prenatal or postpartum exposure to tobacco smoking and allergic rhinitis. This causal relationship is also supported by a number of studies^24,27-29^. Such a relationship is observed with active^30-32^ and passive^27,33^ smoking. The generalizability of our findings to populations in developed countries was supported by broadly consistent results since our data were derived mostly from high-income countries. However, we could not conclude that such findings would be replicated in low-income and middle-income countries because subgroup analysis with adjustment showed that potential confounders might influence the causal association between postpartum smoke exposure and risk of allergic rhinitis^20,23-25^. The meta-analysis by Zhou et al.^28^ included six cohort studies, two case-control studies, and eight cross-sectional studies, and they showed that maternal smoking exposure, especially passive smoking, during pregnancy increased the risk of allergic rhinitis in offspring. Moreover, subgroup analysis showed that active smoking during pregnancy was significantly correlated with offspring allergic rhinitis in cross-sectional studies and studies performed in America^28^. Of course, many smoke compounds cross the placental barrier and can affect the development of the fetus, particularly the immune system^34-36^. Exposure to secondhand smoke after birth will have similar impacts on the development of the respiratory and immune systems^37,38^. Still, the exact mechanisms are complex and were outside the scope of the present meta-analysis.

### Limitations

Notwithstanding the inherited limitations of the included studies, some caveats should be considered while interpreting the results. First, this meta-analysis might suffer from flaws due to marked heterogeneity. The characteristics of included studies, including study population, exposure, and adjustment of variables, might explain part of the heterogeneity. Second, although publication bias was not assessed due to the limited number of included studies, it was unlikely to be observed according to the visualized distribution of studies in the funnel plots. Thirdly, we did not extract the details of exposure because we could not differentiate active smokers from passive smokers, determine the dosage of cigarettes, whether non-smokers included ex-smokers or all of them were never exposed to smoke and the duration of quit of ex-smokers. This prevented further exploration of the dose-response relationship between exposure and outcome. Fourthly, since different ages of offspring were investigated in various studies, this meta-analysis could only be roughly divided into the group <10 years and the group >10 years. The age was the time when the offspring were investigated, but not the age at which allergic rhinitis appeared in the offspring. The time relationship between etiology and outcome can be further analyzed if the original study can provide data on time from onset of symptoms to diagnosis. Future prospective studies and mechanism studies should aim to determine how parental smoking behavior influences the development of allergic rhinitis.

## CONCLUSIONS

Our study suggests that parental smoking exposure during gestation might contribute to the development of allergic rhinitis in offspring, and also postnatal parental smoking exposure is an independent risk factor for allergic rhinitis in their offspring. These finding may influence the public health prevention of allergic rhinitis, indicating the need for protecting pregnant women and young children from cigarette smoke exposure.

## Data Availability

All data generated or analyzed during this study are included in this article and its supplementary material files. Further enquiries can be directed to the corresponding author.
