# Supplementary material for: Association between prenatal or postpartum exposure to tobacco smoking and allergic rhinitis in the offspring: An updated meta-analysis of nine cohort studies
Source: Tob Induc Dis. 2022 Apr 11;20:37. doi: 10.18332/tid/146905 (PMC8996217; doi:10.18332/tid/146905)
Supplement: Supplementary file 1 [file TID-20-37-s1.pdf]

Supplementary Material

Supplementary Figure 1. Sensitivity analysis of the association between allergic rhinitis and prenatal smoke exposure

Supplementary Figure 2. Sensitivity analysis of the association between allergic rhinitis and postpartum smoke exposure

Supplementary Figure 3. Funnel plot for assessment of publication bias in association between allergic rhinitis and prenatal smoke exposure

Supplementary Figure 4. Funnel plot for assessment of publication bias in association between allergic rhinitis and postpartum smoke exposure

Supplementary Table S1 Search strategy

Supplementary Table 2. NOS criteria for quality of cohort study.

Supplementary Table 3. Study and patient characteristics

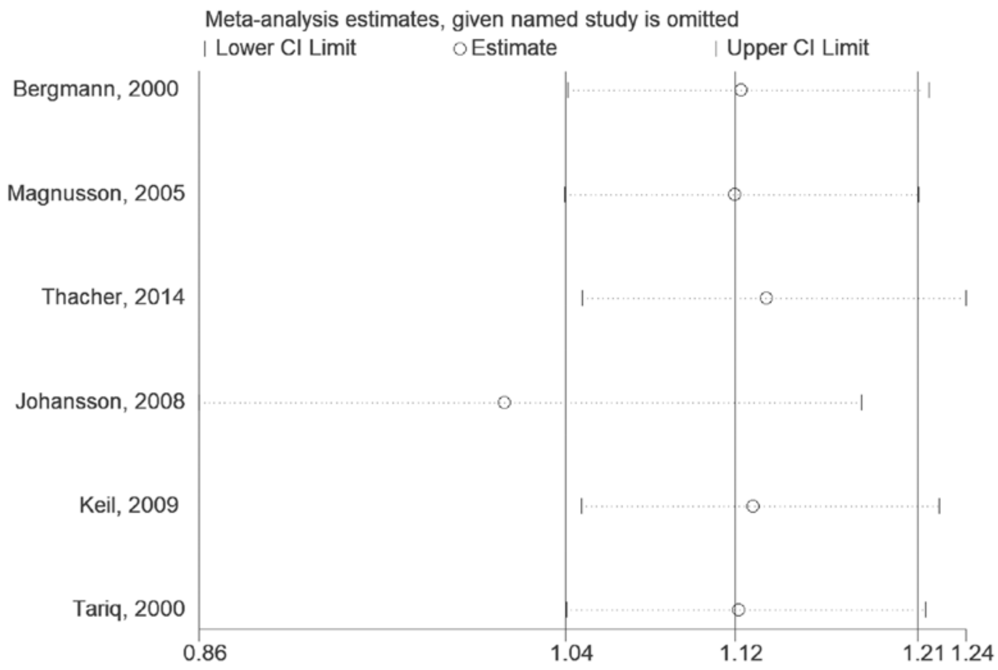

Supplementary Figure 1. Sensitivity analysis of the association between allergic rhinitis and prenatal smoke exposure

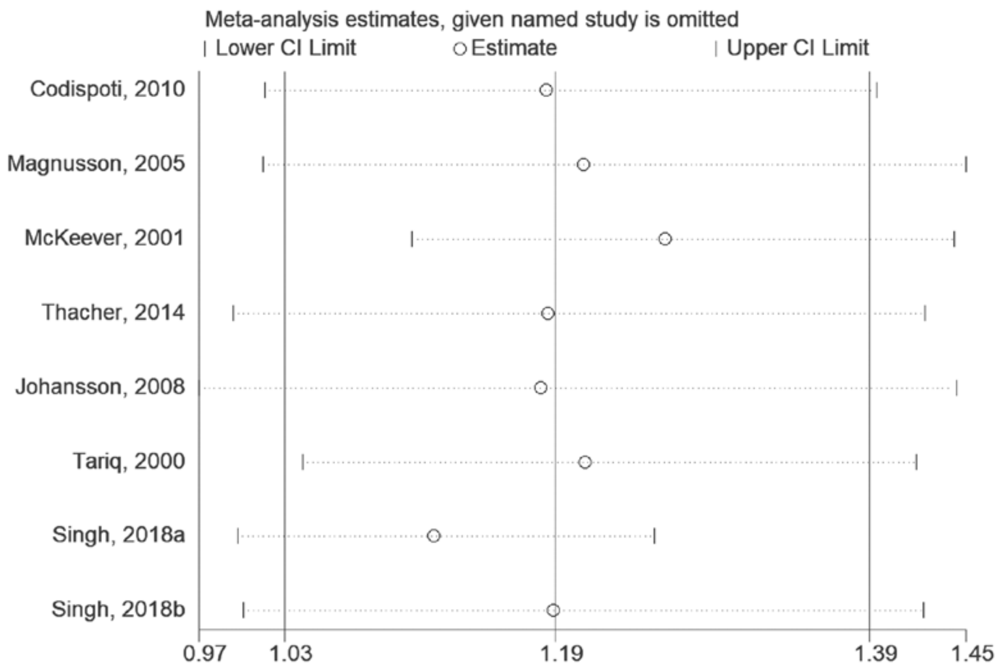

Supplementary Figure 2. Sensitivity analysis of the association between allergic rhinitis and postpartum smoke exposure

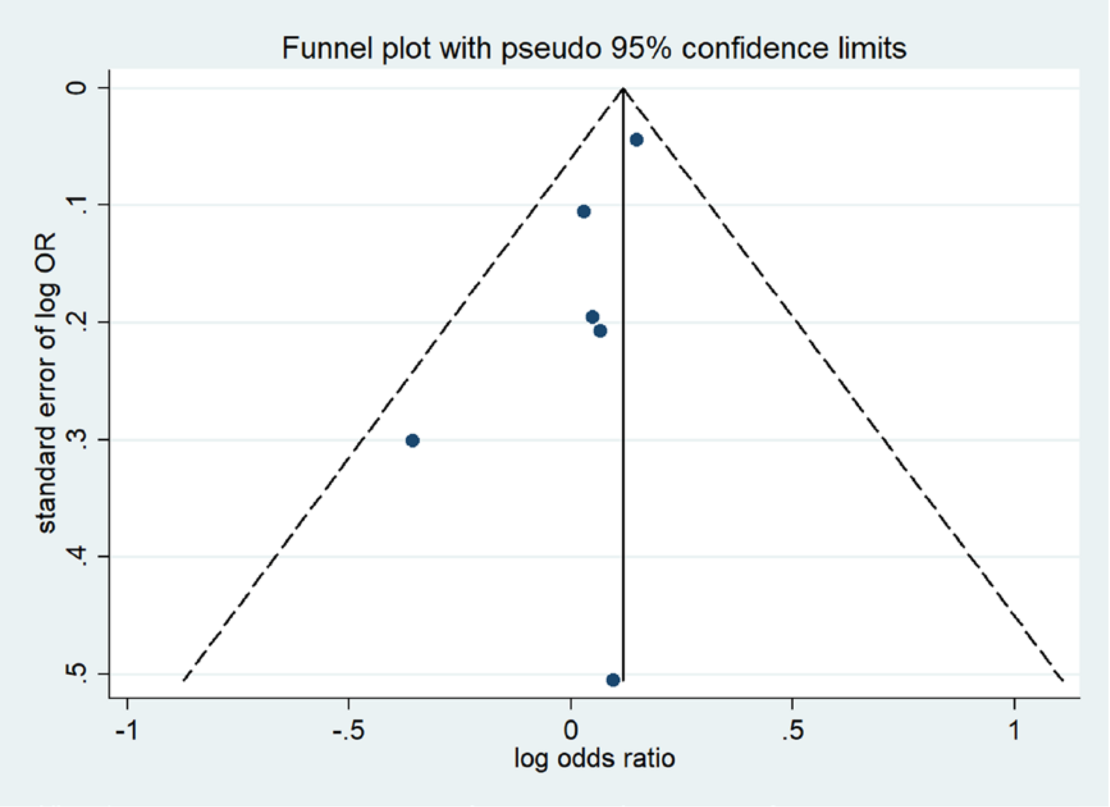

Supplementary Figure 3. Funnel plot for assessment of publication bias in association between allergic rhinitis and prenatal smoke exposure

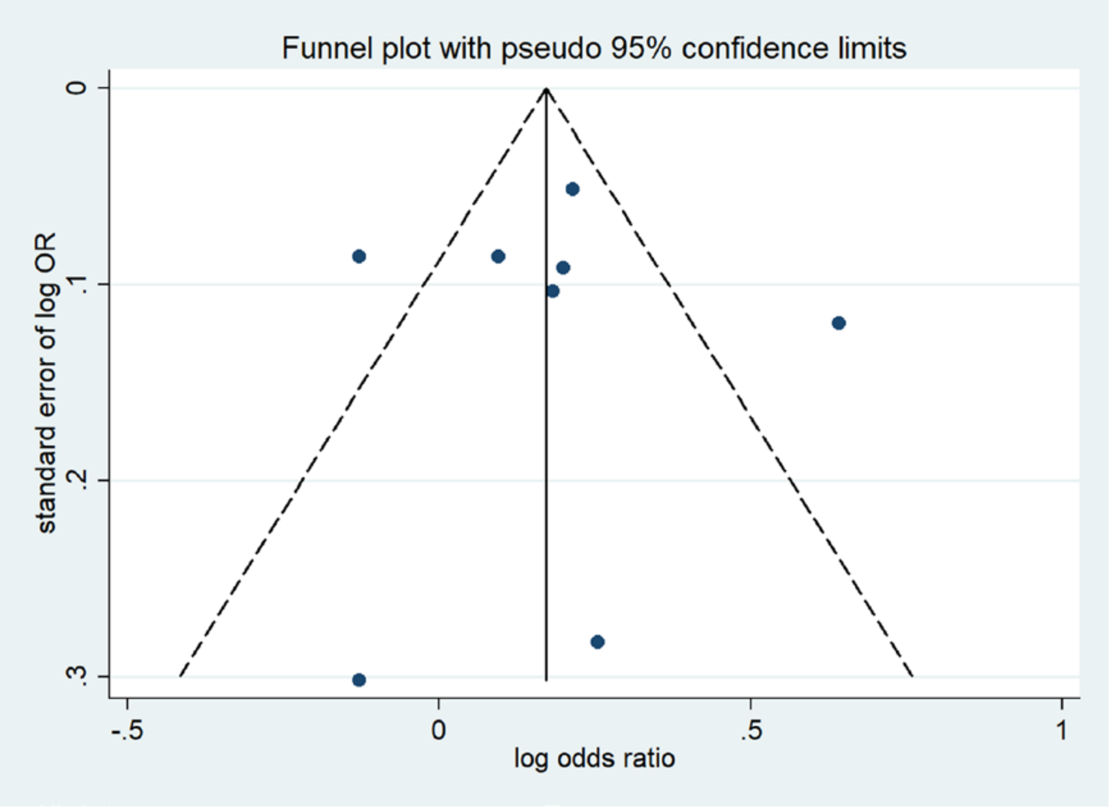

Supplementary Figure 4. Funnel plot for assessment of publication bias in association between allergic rhinitis and postpartum smoke exposure

Supplementary Table S1. Search terms and strategy.

Date:2020/07/20

**PubMed**		**Search strategy**	**Numbers**
**Patient**	**#1**	(maternal[Title/Abstract]) OR (mother*[Title/Abstract]) OR (father*[Title/Abstract]) OR (parent*[Title/Abstract])	784687
**Intervention**	**#2**	"Smoking"[Mesh] OR (Smoking Behaviors[Title/Abstract] OR Behavior, Smoking[Title/Abstract] OR Behaviors, Smoking[Title/Abstract] OR Smoking Behavior[Title/Abstract] OR Smoking Habit[Title/Abstract] OR Habit, Smoking[Title/Abstract] OR Habits, Smoking[Title/Abstract] OR Smoking Habits[Title/Abstract])	156168
	**#3**	("Tobacco Use"[Mesh]) OR (Tobacco Uses[Title/Abstract] OR Tobacco Chewing[Title/Abstract] OR Chewing, Tobacco[Title/Abstract] OR Tobacco Consumption[Title/Abstract] OR Consumption, Tobacco[Title/Abstract])	8925
	**#4**	#2 OR #3	159466
**Outcome**	**#5**	((((((((child[Title/Abstract]) OR (children[Title/Abstract])) OR (teen*[Title/Abstract])) OR (adolescent[Title/Abstract])) OR (infant[Title/Abstract])) OR (newborn[Title/Abstract])) OR (offspring[Title/Abstract])) OR (childhood[Title/Abstract])) OR (preschool[Title/Abstract])	1790788
	**#6**	((("Rhinitis, Allergic"[Mesh]) OR (Allergic Rhinitides[Title/Abstract] OR Rhinitides, Allergic[Title/Abstract] OR Allergic Rhinitis[Title/Abstract]) OR "Rhinitis, Allergic, Seasonal"[Mesh]) OR (Seasonal Allergic Rhinitis[Title/Abstract] OR Allergic Rhinitides, Seasonal[Title/Abstract] OR Allergic Rhinitis, Seasonal[Title/Abstract] OR Rhinitides, Seasonal Allergic[Title/Abstract] OR Rhinitis, Seasonal Allergic[Title/Abstract] OR Seasonal Allergic Rhinitides[Title/Abstract] OR Pollen	33150

		Allergy[Title/Abstract] OR Allergies, Pollen[Title/Abstract] OR Allergy, Pollen[Title/Abstract] OR Pollen Allergies[Title/Abstract] OR Pollinosis[Title/Abstract] OR Pollinoses[Title/Abstract] OR Hay Fever[Title/Abstract] OR Fever, Hay[Title/Abstract] OR Hayfever[Title/Abstract]) OR "Rhinitis, Allergic, Perennial"[Mesh]) OR (Rhinitis, Allergic, Nonseasonal[Title/Abstract])	
	**#7**	#5 AND #6	6684
**Study type**	#8	(cohort) OR (follow?up stud*) OR (longitudinal stud*) OR (odds ratio) OR (relative?risk) OR (rate?ratio) OR (hazard ratio) OR (prospective) OR (retrospective)	4978211
**ALL**	**#9**	#1 AND #4 AND #7 AND #8	81

Embase		Search strategy	Numbers
**Patient**	**#1**	'maternal':ab,ti OR 'mother*':ab,ti OR 'father*':ab,ti OR 'parent*':ab,ti	994953
**Intervention**	**#2**	'smoking'/exp OR 'smoking behaviors':ab,ti OR 'behavior, smoking':ab,ti OR 'behaviors, smoking':ab,ti OR 'smoking behavior':ab,ti OR 'smoking habit':ab,ti OR 'habit, smoking':ab,ti OR 'habits, smoking':ab,ti OR 'smoking habits':ab,ti	397080
	**#3**	'tobacco use'/exp OR 'tobacco uses':ab,ti OR 'tobacco chewing':ab,ti OR 'chewing, tobacco':ab,ti OR 'tobacco consumption':ab,ti OR 'consumption, tobacco':ab,ti	402445
	**#4**	#2 OR #3	407110
**Outcome**	**#5**	'allergic rhinitis'/exp OR 'allergic rhinitides':ab,ti OR 'rhinitides, allergic':ab,ti OR 'allergic rhinitis':ab,ti OR 'pollen allergy'/exp OR 'perennial rhinitis'/exp OR 'seasonal allergic rhinitis':ab,ti OR 'allergic rhinitides, seasonal':ab,ti OR 'allergic rhinitis, seasonal':ab,ti OR 'rhinitides, seasonal allergic':ab,ti OR 'rhinitis, seasonal allergic':ab,ti OR 'seasonal allergic rhinitides':ab,ti OR 'pollen allergy':ab,ti OR 'allergies, pollen':ab,ti OR 'allergy, pollen':ab,ti OR 'pollen allergies':ab,ti OR 'pollinosis':ab,ti OR 'pollinoses':ab,ti OR 'hay fever':ab,ti OR 'fever, hay':ab,ti OR 'hayfever':ab,ti OR 'rhinitis, allergic, nonseasonal':ab,ti	52869
	#6	'child'/exp OR 'child' OR 'children':ab,ti OR 'teen*':ab,ti OR 'adolescent':ab,ti OR 'infant':ab,ti OR 'newborn':ab,ti OR 'offspring':ab,ti OR 'childhood':ab,ti OR 'preschool':ab,ti	3804059
	#7	#5 AND #6	14736
**Study type**	#8	cohort OR 'follow?up stud*' OR 'longitudinal stud*' OR 'odds ratio' OR relative?risk OR rate?ratio OR 'hazard ratio' OR prospective OR retrospective	3142178
**Total**	#9	#1 AND #4 AND #7 AND #8	191

Supplementary Table 2. NOS criteria for quality of cohort study.

Study	Representativeness of the exposed cohort	Selection of the non-exposed cohort	Ascertainment of exposure	Demonstration that outcome of interest was not present at start of study	Comparability of cohorts on the basis of the design or analysis	Assessment of outcome	Was follow-up long enough for outcomes to occur	Adequacy of follow up of cohorts	Total quality scores
Bergmann, 2000	☆	☆	☆	☆	☆	☆	☆	☆	8
Codispoti, 2010	☆	/	☆	☆	☆	☆	☆	/	7
Magnusson, 2005	☆	☆	☆	/	☆	☆	☆	☆	7
McKeever, 2001	☆	/	☆	☆	☆	☆	☆	☆	7

Thacher, 2014	☆	☆	☆	/	☆	☆	☆	☆	7
Johansson, 2008	☆	☆	☆	☆	☆	☆	☆	☆	8
Keil, 2009	☆	☆	☆	☆	☆	☆	☆	☆	8
Tariq, 2000	☆	☆	☆	/	☆	☆	☆	☆	7
Singh, 2018	☆	☆	☆	☆	☆☆	☆	☆	☆	9

Supplementary Table 3. Study and patient characteristics

Study	Country	Number	Age of assessing	Sources of exposure	OR (95%CI)	Variables of Adjustment,Matching, or Restriction	Type of active or passive	Diagnosis	Number of cigarettes
Bergmann, 2000	Germany	825	3-6 y	Mother	pre-:1.05 (0.72–1.55)	Age, sex, parental atopy, socioeconomic status, breast feeding, aeroallergen and food sensitivity, study center	Active	Questionnaires, and had multi-allergen screening tests for sensitization	/

Codispoti, 2010	USA	361	3 y	Parents	post-: 1.29 (0.74–2.24)	Unadjusted	Passive	House dust samples	/
Magnusson, 2005	Denmark	7844	14-18 y	Parents	pre-:1.1(0.4-2.9) post-:1.1(1.0-1.4)	Socio-economic group, maternal occupation, maternal age in pregnancy, coffee consumption in pregnancy, parity, breastfeeding, gender	Passive	Questionnaires	/
McKeever	UK	29238	Up to 6	Mother	post-:0.88(0.75-1.05)	Unadjusted	Active	/	/

, 2001			y						
Thacher, 2014	Sweden	3798	Up to 16 y	Mother	pre-: 1.03 (0.84-1.27) post-:1.22 (1.02-1.46)	Parental smoking throughout childhood	Active	Questionnaires	Above 1 cigarette /day
Johansson, 2008	Sweden	8850	3 y	Mother or both parents	pre-:1.16 (1.06–1.26) post-1.24 (1.12-1.37)	Age, mothers' education, family type	Active and Passive	Questionnaires	/
Keil, 2009	Germany	784	10 y	Parents	pre-: 0.7 (0.4–1.3)	Age, sex, birth weight, breast feeding, siblings, pets, parental education, IgE, location	Passive	Questionnaires	/

Tariq, 2000	UK	1218	4 y	Mother	pre-: 1.07 (0.79–1.78) post-: 0.88 (0.49, 1.60)	Age	Active	Recorded at recruitment	/
Singh, 2018a	India	44928	6-7y	Parents	post-: Mother:1.9 (1.5-2.4), Father:1.4 (1.2-1.5)	Unadjusted	Passive	Environmental questionnaire	/
Singh, 2018b	India	48088	13-14y	Parents	post-: Mother:1.2 (1.0-1.5), Father:1.0 (0.9-1.1)	Unadjusted	Passive	Environmental questionnaire	/

© 2022 Li X. et al.
